# Efficacy and safety of duloxetine and Pregabalin in Iranian patients with diabetic peripheral neuropathic pain: a double-blind, randomized clinical trial

**DOI:** 10.1007/s40200-019-00427-w

**Published:** 2019-08-13

**Authors:** Khojasteh Joharchi, Moosareza Memari, Eznollah Azargashb, Navid Saadat

**Affiliations:** 1 Department of Pharmacology, School of Medicine, Shahid Beheshti University of Medical Sciences (SBUMS), Tehran, Iran; 2 Department of Social Medicine, Faculty of Medicine, Shahid Beheshti University of Medical Sciences, Tehran, Iran; 3 Research Institute for Endocrine Sciences, Shahid Beheshti University of Medical Sciences, Tehran, Iran

**Keywords:** Adverse drug reactions (ADRs), Diabetes, Diabetic peripheral neuropathic pain (DPNP), Duloxetine (DLX), Pregabalin (PGB)

## Abstract

**Purpose:**

Diabetic peripheral neuropathic pain (DPNP) is one of the most sufferings, disabling, and dominant complications of diabetes. Duloxetine (DLX) and Pregabalin (PGB) are among first-line therapy and the most prescribed drugs for DPNP relief. The effectiveness-risk profile of drugs may differ from region to region due to variations in genetic and health situation of populations. This study aims to evaluate the efficacy and safety of DLX and PGB in a sample of Iranian population with DPNP.

**Methods:**

A double-blind, randomized clinical trial was conducted on 180 type-2 diabetic patients with DPNP≥40 mm according to Visual Analogue Scale (VAS), with other eligibility criteria throughout twelve weeks. We divided the patients randomly into two equal groups: DLX and PGB. Each patient received ten days placebo as a washout period, then blind capsules of DLX (group 1) or PGB (group 2). We assessed the efficacy and safety of drugs by VAS and recorded the Adverse Drug Reactions (ADRs) during the study.

**Results:**

In the DLX group, sixty-six and the PGB group, seventy-eight patients completed the study. The intensity of patients’ pain was improved by both drugs significantly (p˂0.001), but there was no significant difference between the two groups. Average daily doses of DLX and PGB were 42.5 and 235.5 mg, respectively. In the DLX group, 74% of patients and the PGB group, 37% reported ADRs. The discontinuation rates due to ADRs were 19% and 7% correspondingly.

**Conclusion:**

We found that in Iranian patients, the mean effective doses of these drugs are different in comparison with several other studies. Surprisingly intolerance and discontinuation of DLX in our patients were attributed to mild and severe Serotonin Syndrome, which had not much occurred in other studies. Accordingly, despite the same efficacy, PGB was better tolerated than DLX in our patients. Thus we would recommend PGB for DPNP treatment in Iranian patients.

## Introduction

Nearly 4.5 million Iranians were suffering from diabetes in 2011 [1], and this number is expected to rise to 9.5 million by 2030 [2]. The prevalence of DPNP in patients with diabetic neuropathy was reported about 11%, 20% and 50% from the United States, England, and the Middle East, respectively [3–5]. Some studies estimated that about 30% - 50% of Iranian diabetic patients have peripheral neuropathy [6, 7]. But we did not found any reports about the prevalence of DPNP in Iranian diabetic patients; however, regarding this high incidence of peripheral neuropathy, it can be estimated that a high proportion of Iranian diabetic patients are suffering from DPNP.

On the Other hand, DPNP has adverse effects on health and quality of life and also causes expensive costs for both patients and health systems of all countries, including Iran. Furthermore, in contrast to nociceptive pain, DPNP is a more frustrating pain, which leads to persistent inconvenience, depression, and abandonment of daily activities.

The characteristics of DPNP are three distinct types of pain, including dysesthesia, paresthesia, and muscle electrical shock-like pain. The patients may also experience allodynia and hyperalgesia and severe painful coldness. These symptoms begin at the lower extremities and develop to hands by disease progression and are known as the model of stocks and gloves. The pain gets worse during the night, and origins sleep problems. The patient’s activity decreases during the day by a sense of walking on the sand and rock. Consequently, the disturbances of night sleep and reduction of daily activity will deteriorate the patients’ blood glucose and progress diabetes [8].

There are some pharmacological and non-pharmacological methods for treating DPNP. In some cases, control of blood glucose can improve DPNP. In other patients, specific modes of electrical stimulation of nerves [9], acupuncture [10], and other non-pharmacological treatments may relief DPNP. However, the pharmacotherapy of DPNP contains serotonin-norepinephrine reuptake inhibitors such as DLX, alpha-2 delta calcium channel antagonists, like PGB, tricyclic antidepressants, opioids, and topical analgesics [11]. The FDA (Food and Drug Association of America) recommended either DLX or PGA as the first-line therapy of DPNP, and they are also approved for this usage in Iran. The effectiveness and risk profile of drugs may differ from region to region due to the variations in genetic and health situations of populations [12, 13].

There have not been any studies about the comparison of efficacy, dosage pattern, and safety of DLX and PGB in Iranian patients with DPNP. Consequently, we conducted this clinical trial to compare the effectiveness and safety of DLX and PGA for DPNP treatment in a sample of Iranian patients.

## Materials and methods

### Patients

This study was conducted according to the Helsinki Declaration and was approved by our University Ethics Committee and also was registered in the Iranian registry of clinical trials.

We selected the volunteers from two diabetic clinics of our University of Medical Sciences through some inclusion and exclusion criteria.

The inclusion criteria comprised of: 1- Type-2 diabetes (diagnosed and confirmed by an endocrinologist according to American Diabetes Association guideline 2017 [14]; 2- Diabetes duration≥5 years 3- DPNP identified by Michigan Neuropathy Screening Instrument (MNSI) examination which the previous studies validated it [15, 16]; 4- DPNP severity≥40 mm of 11 points (0 = no pain to 10 = severe pain) on Visual Analogue Scale (VAS); 5- pain duration ≥12 months; 6-age ≥ 40and ≤ 65 years; 7-signing informed consent form.

The exclusion criteria included: 1- MNSI examination score < 2; 2- history of any hypersensitivity to DLX or PGB; 3- any hepatic, heart and renal failure; 4- hemoglobin A1c > 11 mg%; 5- vision disability; 6- intellectual disorders; 7- usage of any other analgesics; 8- consumption of any anti-inflammatory or serotonergic drugs within 14 days prior to the beginning of the study; 9- uncontrolled hypertension; 10- smoking; 11- having severe depression and psychological disorders; 12- epilepsy or any other neuropathies; 13- pain attributable to different reasons.

Medications necessary for diabetes control and any other vital drugs such as antihypertensive and anti-dyslipidemia were almost unchanged during the study period.

### Study design

We designed a controlled clinical trial with 12-weeks double-blind, randomized, parallel groups, and conducted it on 180 eligible patients from July 2017 to March 2018. We calculated the sample size by using formula suggested for randomized clinical trials, considering the type I error of 5% (α = 0.05), type II error of 20% (β = 0.2; power = 80%) plus 20% dropout. We divided the patients randomly into two equal groups of DLX and PGB.

We prepared some similar empty capsules and filled them with DLX or PGB or Starch as a placebo. At first, the patients in the DLX group received placebo capsules once daily for ten days as a washout period. Then they had a fixed dose of 30 mg/d of DLX in the first week of treatment and a different dose of 30 to 60 mg/d for the next eleven weeks based on drug efficacy and tolerability. The patients in the PGA group were given placebo twice daily (Bd) for ten days as a washout period, then a fixed dose of 75 mg/Bd in the first week and 75 to 150 mg/Bd for the last eleven weeks. After these twelve weeks and at the end of our study, according to the patients’ pain recovery and their tolerability, the drugs were continued or tapered and switched to other medications.

We assessed the efficacy and safety of the drugs by recording daily phone conversations with patients and monthly physical examinations. VAS measured the intensity of patients’ pain. The patients and also the physicians who determined the VAS value and the adverse drug reactions (ADRs) were both blinded to the kinds of drugs during the study. The rates of ADRs determined the safety of the drugs, and the discontinuation of the treatment was due to the severity of ADRs.

### Statistical analysis

The SPSS software, version 19.0, was used for all statistical analyses, and the *p* value˂0.05 was considered significant. The Kolmogorov-Smirnov test assessed the normality of the data. The mean values of demographic and biochemical characteristics of patients, the monthly intensity of DPNP, and also the incidence of ADRs between the two groups were compared by t-test. The time × treatment interaction and the comparison of mean intensity in every month with the previous month in both groups were analyzed by repeated measure analysis. The quantitative and qualitative variables were reported respectively as mean ± SD, and pure frequency plus its percentage.

## Results

### The consort chart

**Fig. 1:**
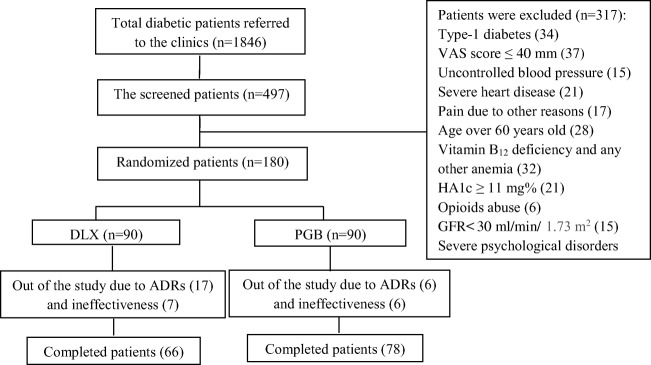
**The consort chart of the patients:** n: number, DLX: Duloxetine, PGB: Pregabalin, ADRs: Adverse Drug Reactions

The flow diagram of studied patients is shown in Fig. 1. A total number of 1846 diabetic patients were interviewed, and 497 patients with peripheral pain were examined in two different diabetic clinics. From these screened patients, 317 were excluded. Finally, 180 patients (71 men and 109 women) were selected according to inclusion and exclusion criteria and randomly divided into two equal groups: DLX and PGB. In the DLX group, 66 patients (73%) and the PGB group, 78 patients (87%) completed the study (Fig. 1).

### The clinical characteristics of the patients

The demographic and primary clinical data of all patients in the two groups are shown in Table 1.

**Table 1:** Mean demographic and clinical characteristics of patients

Characteristics	Intervention groups Mean (SD)		p value
	DLX (*N* = 66)	PGB (*N* = 78)	
Gender			
Male (N)	27	29	
Female (N)	39	49	
Age (Y)	54.93(3.70)	54.03(4.46)	0.388
BMI (*kg*/*m*^*2*^)	26.12(1.02)	26.55(0.99)	0.595
HA1c (mg %)	8.9 (1.20)	8.7(1.72)	0.655
FBG (mg/dl)	146.34 (13.39)	144.74(19.44)	0.699
MNSI questionnaire	6.65 (1.80)	6.71(2.03)	0.810
MNSI examination	2.69 (0.55)	2.82(0.43)	0.076
VAS (mm)	67.23(19.29)	61.74(16.34)	0.052
Serum Creatinine (mg/dl)	0.95(0.11)	1.02(0.09)	0.088
Duration of Diabetes (Y)	9.57(3.20)	9.05(2.85)	0.145
Duration of DPNP (Y)	3.55(1.66)	4.09(2.02)	0.067

SD, Standard Deviation; N, number of patients in groups that completed the study; Y, years; BMI, Body Mass Index; FBG, Fasting Blood Glucose; MNSI, Michigan Neuropathy Scale Instrument; VAS, Visual Analogue Scale

### The dose profile of the drugs

We found the average daily doses of DLX and PGA during 12 weeks era of the study to be 42.5 and 235.5 mg, respectively.

### The DPNP intensity in both groups

**Fig. 2:**
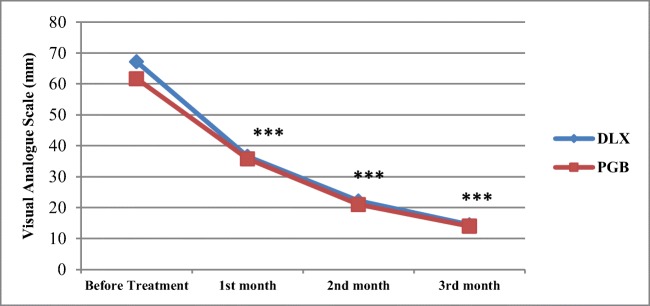
Visual Analogue Scale score at different time sections of the study in both groups (time× drug interaction curves)***,*** Mean VAS score of monthly time sections in each group *** p value˂0.001

The mean DPNP intensities of both groups according to the VAS score, in different months, are presented in Fig. 2. Repeated measure analysis exhibited that in both drugs, the time × drug interaction in all time sections of the study was significant (P ˂ 0.001). The comparison of DPNP intensities in each group at monthly time sections of the study is shown in Table 2. In both DLX and PGB group, the differences between DPNP intensity at each month versus the previous month were significant (p˂0.001). But as it is evident in Fig. 2, there were no significant differences in DPNP intensities between the two groups in any time section of the study.

**Table 2:** Comparison of patients’ DPNP intensity, mean ± (SD) at time sections of the study

DPNP mean by VAS (mm)		p- value
DLX		
Before treatment	1st- month	
67.2(19.3)	32.4(8.5)	˂0.001
1st-month	2nd- month	
32.4(8.5)	22.3(6.4)	˂0.001
2nd-month	3rd-month	
22.3(6.4)	16.2(4.2)	˂0.001
PGA		
Before treatment	1st- month	
61.7 (16.3)	29.7(7.8)	˂0.001
1st-month	2nd-month	
29.7(7.8)	22.1(6.4)	˂0.001
2nd-month	3rd-month	
22.3(6.4)	16.0(5.5)	˂0.001

SD: Standard Deviation; mm: millimeter

### The adverse drug reactions (ADRs) in both groups

In DLX group sixty-seven (74%) of patients and PGA group thirty-four (37%) reported at least one ADR related to the medications. The rate of ADRs emerged in patients are demonstrated in Table 3. There are meaningful differences between the two groups regarding the incidence rate of some ADRs (p˂0.05, =0.01 & ˂0.001).

**Table 3:** ADRs of DLX versus PGA in patients

ADRs	DLX (N)	PGB (N)	p value
Anorexia	22(25%)	2(2%)	˂0.001
Nausea	20(23%)	2(2%)	˂0.001
Vomiting	10(11%)	0(0%)	˂0.001
Shivering	16(18%)	0(0%)	˂0.001
Agitation	14(16%)	0(0%)	˂0.001
Tremor	14(16%)	0(0%)	˂0.001
Muscle rigidity	14(16%)	0(0%)	˂0.001
Diaphoresis	11(12%)	0(0%)	˂0.001
Abdominal cramp	14(16%)	2(2%)	0.02
Diarrhea	10(11%)	3(3%)	0.02
Hyperthermia	8(9%)	0(0%)	˂0.001
Headache	4 (5%)	6(7%)	0.75
Hypertension	10(11%)	2(2%)	0.01
Tachycardia	10(11%)	2(2%)	0.01
Arrhythmia	4(5%)	0(0%)	0.06
Increased micturition	8(9%)	1(1%)	0.01
Weight gain	0(0%)	16(18%)	˂0.001
Dizziness	4(5%)	14 (15%)	0.06
Somnolence	8(9%)	18(20%)	0.06
Edema	0(0%)	11(12%)	˂0.001

N, number of patients encountered ADRs

**Fig. 3:**
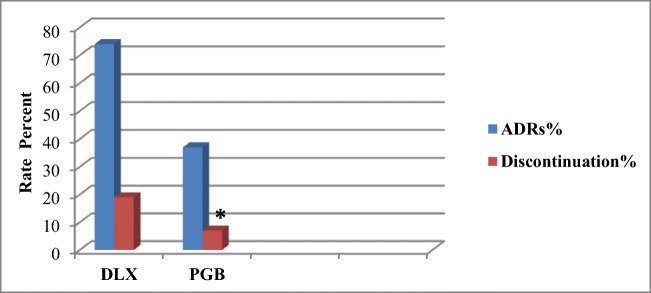
The Percentages of ADRs and discontinuation rates as a result of ADRs in both groups of DLX and PGB; *p < 0.05

Several ADRs in each group were mild and somehow tolerable, but certain others were severe and intolerable that caused discontinuation of the drugs. The discontinuation rates due to ADRs in the DLX and PGA groups were 19% and 7% individually, which showed a significant difference between the two groups (p˂0.05). The percentages of ADRs and discontinuation rates attributable to ADRs in patients of both groups are exhibited in Fig. 3.

## Discussion

In this clinical trial, we compared the efficacy and safety of DLX and PGB in a sample of Iranian patients with DPNP for the first time. The monthly DPNP intensity of patients who tolerated the drugs in comparison to the previous month was significantly relieved in both groups, which are presented in Fig. 2. But according to the VAS score, there was no significant difference in efficacy of DLX compared with PGA along with the study. This result is dependable with several previous studies that examined this issue in other populations [17–24]. Among these studies, Boyle et al. [17], Tanenberg et al. [18], Devi et al. [19], compared the two drugs directly and the study of Quilici et al. [20] was a meta-analysis of nine types of research. In all these studies, parallel to our study, the DPNP relief effect of DLX and PGB did not differ significantly.

Furthermore, in our study, the prescribed dose of each drug was flexible via patients’ responsiveness. The average daily dose obtained for DLX in Iranian patients was 42.5 mg/d, which was beneath several related studies in other populations [17, 18, 20, 22, 25, 26]. The average daily therapeutic doses of DLX in two non-interventional Post-Hoc studies were 53.9 and 55.2 mg/d in German and American patients with DPNP [25, 26], which were higher than our study. Boyle et al. reported the higher doses of DLX (60 and 120 mg/d) on patients with DPNP in the United Kingdom, which both treatments were efficient without significant difference [17]. Tanenberg et al. conducted a similar study on white men from worldwide countries and reported a suitable dose of 60 mg/d for DLX [18].

Moreover, Devi et al. directed a clinical trial about the efficacy and safety of DLX and PGB in Indian patients with DPNP and prescribed flexible doses of 20 to 80 mg/d DLX via dose-responses [19]. In another clinical trial, Zakerkish et al. recommended flexible doses of 30 to 60 mg/d DLX in comparison to Nortryptilene for the patients with DPNP in Ahvaz-south of Iran [27]. Zakerkish et al. and Devi et al. did not report the average prescribed doses of drugs in their studies. The average dose of DLX in our study was almost similar to the study of Yasuda et al. in Japanese patients with DPNP that were given a fixed dose of 40 mg/d [21]. Another similarity between our study and Yasuda et al. was the average weight of patients (60 kg). Thus the resemblance of doses may be somehow related to the similarity of average weights. In the study of Raskin et al. which the average weight of patients (82 kg) was higher than our study, the effective dose of DLX was 120 mg/d that may be somewhat related to the higher weight of the patients [22]. But, in the other above mentioned articles, the average weight of patients had not been reported so we could not do further analysis of the correlation between weight and the dose of DLX.

The average daily dose of PGB in the present study was 235.5 mg/d, which is higher than 173.5 mg/d as reported by Happich et al. [25]. However, the average daily dose of PGB in our study in comparison with three other kinds of research [17, 18, 24] was lower. The PGB doses in the study of Devi et al. were flexible from 150 to 600 mg/d [19] but in the studies of Boyle et al. [17] and Tanenberg et al. [18] were fixed doses of 600/d and 300 mg/d respectively, which all are above the average dose of PGB in this study.

On the other hand, we found the ADRs of PGB to be almost in line with other previous studies. Some ADRs such as dizziness, weight gain, drowsiness, and edema emerged in PGB group was significantly more than the DLX group (Table 3). These ADRs were similar to previous studies [17–19], but surprisingly in this study, different results about kinds of ADRs of DLX were observed. In several various studies significant reported ADRs in DLX groups included anorexia, nausea, vomiting, and diaphoresis [17–22, 27], but tremor, shivering, muscle rigidity, agitation, hypertension, tachycardia that observed in a percentage of our patients were not reported that much by similar previous studies (Table 3). The emerged ADRs about DLX group in this clinical trial plus diaphoresis, hyperthermia, nausea, vomiting according to Sternbach and Hunter criteria are signs and symptoms of Serotonin Syndrome (SS). The Sternbach and Hunter criteria that we used to detect SS have been recognized as two standard procedures for assessing SS [28]. For differential diagnosis of SS, we excluded similar conditions such as neuroleptic malignant syndrome, malignant hyperthermia, sympathomimetic intoxication, anticholinergic intoxication, sedative-hypnotic withdrawal, meningitis, and encephalitis by the history of associated drugs use and clinical examination. The major causes of DLX discontinuation in our trial were moderate to severe SS. Although arrhythmia incidence in DLX group was not statistically more than PGB group (*P* = 0.06) but due to the importance of this ADR and the different incidence rates in two groups (5% in DLX vs. 0%in PGB), it would be clinically significant.

**Fig. 4:**
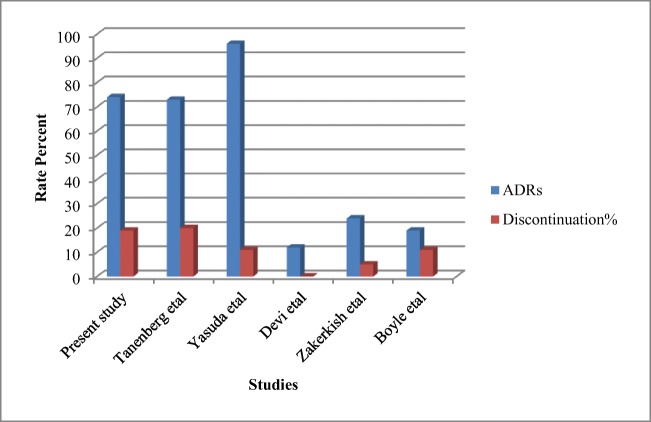
The rates of DLX Adverse Drug Reactions (ADR)% and Discontinuation % in the present study and some other studies

In addition to the kinds of ADRs, there were differences in rates of ADRs and discontinuation of DLX in our study as well as other studies. In this study, the ADRs and discontinuation rates were 74% and 19% about DLX, also 37% and 7% about PGB. But Devi et al. reported ADRs rates of DLX and PGB to be 9% entirely, and none of the patients discontinued the study for the reason of ADRs. According to Zakerkish et al., the ADRs of DLX versus Nortryptilene was negligible and did not result in the discontinuation of the trial [27]. Moreover, Yasuda et al. reported the rates of 96% ADRs and 22% discontinuation [21]. In the study of Boyle et al. [17], discontinuation rate of PGB group was higher than DLX group (22% vs. 11%) in contrast to our study (7% vs. 19%) and Tanenberg et al. study [18] was(10% vs. 19.6%), somehow similar to this study. The differences in rates of ADRs and discontinuation of DLX in different studies are presented in Fig. 4.

One of the possible explanations for different effective dose and safety of DLX in different studies may be the gene polymorphism. The specific pharmacodynamics and pharmacokinetic profiles of patients related to genotypic differences can cause diverse efficacy and safety drug profiles in various populations [29]. In 2007 in an animal model, Fox et al. reported that polymorphism in the serotonin transporter gene creates susceptibility to SS [30]. On the other hand, DLX elimination is chiefly done by hepatic metabolism. Two enzymes, CytochromeP1A2 (CYP1A2) and Cytochrome2D6 (CYP2D6), are involved in hepatic metabolism of DLX [31, 32]. In some reports, patients with CYP1A2 gene polymorphism suffered from severe ADRs of antipsychotic and anti-rheumatic drugs [33, 34]. There is a report that 24 % of Iranians have the specific gene of CYP2D6, which makes them relevant poor metabolizer. These patients suffer from ADRs of antipsychotics and antidepressants [35]. We can conclude that CYP1A2 and CYP2D6 gene polymorphism may exist in our patients who encountered ADRs.

The other factors such as social or natural environments, mental condition, nutrition, and hypoalbuminemia may also cause differences in dose-response and ADRs in different populations and regions [36, 37] which the future investigations will clarify the probable role of each one of these factors.

In conclusion, despite the equal efficacy of DLX and PGB, it seems that PGB is well tolerated than DLX for the treatment of DPNP in Iranian patients. In a different population, different dosage recommendations of DLX and PGB may be required for individual patients. Similarly, some clinical practices had recommended the lower doses of other certain drugs such as Tricyclic Antidepressants and Warfarin to reach a suitable effective dose in Iranian patients compared to Europeans and Americans [38]. Thus we highly recommend PGB rather than DLX for DPNP relief in Iranian diabetic patients, and in exceptional cases, if there is a force to use DLX, lower doses should be prescribed with extreme care for SS and probable arrhythmia.

## Limitations and suggestions of the study

The Committee on Medical Ethics of our University objected having a long time placebo control group due to ethical issues in patients having pain. Thus we did not have a control group. However, each drug receiving group was considered as an active control group for another one.

As a recommendation, studies on the discovery of genetic polymorphism will help determine dosage regimens and ADRs before prescribing the medications.

Another suggestion is to design studies about the effect of other factors, such as social and natural environment, mental condition, and nutrition on efficacy and ADRs of drugs.
