# Correction to: Efficacy and safety of duloxetine and Pregabalin in Iranian patients with diabetic peripheral neuropathic pain: a double-blind, randomized clinical trial

**DOI:** 10.1007/s40200-019-00440-z

**Published:** 2019-09-13

**Authors:** Khojasteh Joharchi, Moosareza Memari, Eznollah Azargashb, Navid Saadat

**Affiliations:** 1 Department of Pharmacology, School of Medicine, Shahid Beheshti University of Medical Sciences (SBUMS), Tehran, Iran; 2 Department of Social Medicine, Faculty of Medicine, Shahid Beheshti University of Medical Sciences, Tehran, Iran; 3 Research Institute for Endocrine Sciences, Shahid Beheshti University of Medical Sciences, Tehran, Iran

**Correction to: Journal of Diabetes & Metabolic Disorders**

10.1007/s40200-019-00427-w

The article Efficacy and safety of duloxetine and Pregabalin in Iranian patients with diabetic peripheral neuropathic pain: a double-blind, randomized clinical trial, written by Khojasteh Joharchi, Moosareza Memari, Eznollah Azargashb, and Navid Saadat, was originally published electronically on the publisher’s internet portal (currently SpringerLink) on 13 August 2019 without open access.

With the author(s)’ decision to opt for Open Choice the copyright of the article changed on 12 September 2019 to © The Author(s) 2019 and the article is forthwith distributed under the terms of the Creative Commons Attribution 4.0 International License (http://creativecommons.org/licenses/by/4.0/), which permits use, duplication, adaptation, distribution and reproduction in any medium or format, as long as you give appropriate credit to the original author(s) and the source, provide a link to the Creative Commons license and indicate if changes were made.

